# The Dual Role of Hepcidin in Brain Iron Load and Inflammation

**DOI:** 10.3389/fnins.2018.00740

**Published:** 2018-10-15

**Authors:** Driton Vela

**Affiliations:** Department of Physiology, Faculty of Medicine, University of Pristina, Pristina, Kosovo

**Keywords:** hepcidin, brain, neurodegenerative diseases, glial cells, inflammation, iron load

## Abstract

Hepcidin is the major regulator of systemic iron metabolism, while the role of this peptide in the brain has just recently been elucidated. Studies suggest a dual role of hepcidin in neuronal iron load and inflammation. This is important since neuronal iron load and inflammation are pathophysiological processes frequently associated with neurodegeneration. Furthermore, manipulation of hepcidin activity has recently been used to recover neuronal damage due to brain inflammation in animal models and cultured cells. Therefore, understanding the mechanistic insights of hepcidin action in the brain is important to uncover its role in treating neuronal damage in neurodegenerative diseases.

## Introduction

Iron is a crucial element for maintaining brain homeostasis. It is used by brain cells to synthesize myelin, produce neurotransmitters, and realize a wide range of important cellular biochemical reactions (Ward et al., 2014). But, iron excess is toxic for brain cells. This is observed during brain hemorrhage, where inflammatory signaling is responsible for the increase in brain iron load, which is associated with oxidative damage and cognitive decline (Wu et al., 2003; Ward et al., 2014; Garton et al., 2016). Furthermore, iron dysregulation has been proposed as a pathogenic factor in neurodegenerative diseases as well (Ward et al., 2014). Recent research suggests that brain iron dysmetabolism can be tackled via hepcidin manipulation, since local hepcidin production in the brain has been shown to affect cellular iron transport (Urrutia et al., 2013; Du et al., 2015; Gong et al., 2016; Xiong et al., 2016; Zhou et al., 2017). But, it seems that the presence or absence of inflammation dictates the deleterious vs. beneficial effects of hepcidin in the brain. Therefore, unraveling mechanistic aspects of the dual nature of hepcidin in the brain is important to understand its therapeutic potential.

## Regulation of Systemic Hepcidin and its Actions

Most of systemic hepcidin is produced in hepatocytes via paracrine signaling from liver sinusoidal endothelial cells (LSECs) (Canali et al., 2017). LSECs respond to iron-mediated pathways by producing bone morphogenetic protein 6 (BMP6), which then activates BMP receptor (BMPR) in hepatocytes (Steinbicker et al., 2011). Activated BMPR induces s-mothers against decapentaplegic (SMAD) protein phosphorylation. Phosphorylated SMADs migrate to nucleus where they cause hepcidin upregulation (Casanovas et al., 2009). Although iron-mediated signals act mainly through BMP/SMAD pathway, other pathways are also involved, like mediation through transferrin receptor 2 (TFR2) and hemochromatosis protein (HFE) (Fleming, 2009). The second most studied stimulator of hepcidin expression is inflammation. It induces hepcidin expression via interleukin-6/janus kinase 2/signal transducer and activator of transcription 3 (IL-6/JAK2/STAT3) pathway (Pietrangelo et al., 2007). This pathway does not dominate hepcidin expression in basal conditions, but during high levels of inflammation it can override hepcidin regulation by iron signaling. In addition to these pathways, hepcidin is controlled by negative regulation, which includes signaling via erythroferrone (ERFE), heparin, vitamin D (Poli et al., 2011; Bacchetta et al., 2014; Kautz et al., 2014). Tight regulation of hepcidin production ensures a direct control of iron metabolism. This is attributed to the ability of hepcidin to induce ferroportin (FPN) degradation in target cells (Ramey et al., 2010; Zhang et al., 2011). FPN is known as the major cellular iron exporter, which is the main target protein of hepcidin.

## Regulation of Brain Iron Metabolism

Iron homeostasis in the brain must be finely regulated to serve the purposes of brain cells without damaging cell structures. There are different mechanisms that allow for this to occur; first, blood brain barrier (BBB) is a natural barrier that does not allow passing of iron with ease, which means that iron has to be transported transcellularly via TFR (Bien-Ly et al., 2014; McCarthy and Kosman, 2015a). The iron-TFR complex undergoes endocytosis, while iron gets released intracellularly through divalent metal transporter 1 (DMT1) (Ward et al., 2014). Iron exits from brain cells via FPN (Ward et al., 2014). Higher density of FPN in neurons compared to other brain cells shows why FPN downregulation causes more marked cellular iron load in neurons (Moos and Rosengren Nielsen, 2006; Boserup et al., 2011; Urrutia et al., 2013). Studies suggest that astrocytes encircling brain microvascular endothelial cells (BMVECs) influence their FPN activity by releasing local hepcidin, but also by secreting ferroxidases [including amyloid-β precursor protein (APP)] to stabilize FPN (McCarthy and Kosman, 2014, 2015a,b). In any case, iron released into brain parenchyma enters neuronal cells via TFR1, transient receptor potential canonical (TRPC) channel, DMT1 and newly discovered players, such as Zip8 and Steap2, with DMT1 role being more prominent during pathophysiological processes (Ji and Kosman, 2015; Skjørringe et al., 2015).

## Role of Systemic Hepcidin in Brain Homeostasis

It is believed that systemic hepcidin can cross blood barriers such as BBB (Xiong et al., 2016). Hepcidin is a member of the big family of antimicrobial peptides (AMPs), which have been shown to cross BBB via different pathways (Welling et al., 2015). This argument is further enforced by the ability of a specific group of AMPs named defensins (which includes hepcidin) to cross intact BBB rather easily (Schluesener and Meyermann, 1995). On the other hand, the idea that hepcidin passes intact BBB has been proposed by Raha-Chowdhury et al. (2015). Authors base their conclusion on their observation that hepcidin expression was seen in nearly all cellular layers of BBB, and also due to the fact that protein expression of hepcidin is higher and more robust than its mRNA expression, which has also been corroborated by other studies (Zechel et al., 2006). The idea that hepcidin could pass through intact BBB has been shared by other authors as well (Myhre et al., 2013; Hofer and Perry, 2016). It seems that the amphipathic and cationic chemical structure of defensins in general and hepcidin in particular, might make them potential candidates for BBB pass via transcytosis (Kumagai et al., 1987; Rousselle et al., 2000; Hunter et al., 2002), though this property of hepcidin has still not been conclusively shown in intact BBB. Still, hepcidin passage through BBB is significantly increased with its disruption, and it is observed even with larger peptides than hepcidin (Ostergaard et al., 2009).

It has to be mentioned that models with liver hepcidin knockout do not result in significant brain iron dysmetabolism (Xiong et al., 2016). This would be the reason why we do not observe significant brain iron overload in hemochromatosis (Rutgers et al., 2007). This means that autonomous mechanisms in the brain are responsible for keeping iron metabolism in check. These mechanisms are mostly realized through the activity of glial cells, which are capable of buffering excess iron (Pelizzoni et al., 2013; Codazzi et al., 2015; McCarthy et al., 2018). Astrocytes are especially active during iron transport through BBB (McCarthy and Kosman, 2015b), while microglia are responsible for the control of parenchymal brain iron transport (McCarthy et al., 2018). Microglia are important “managers” of the response to brain damage due to their first-time reaction and commanding role in this respect (Shinozaki et al., 2017). On the other hand, astrocytes are able to transport the much needed iron for neuronal needs as well, which is why conditional deletion of FPN in astrocytes damages the process of myelination (Schulz et al., 2012). FPN is also important for the iron transport through BMVEC, which is why specific BMVEC FPN mutations cause transient brain iron deficiency. This process is reversible due to upregulation of iron import proteins in BMVEC (Iacovelli et al., 2009). This means that significant injury to brain cells from iron overload will occur if both, cellular iron import and export proteins are affected. Cellular iron overload seems to be more detrimental to neurons due to their lower iron buffering capacity (Bishop et al., 2011). But, studies suggest that glial cell beneficial phenotype can also be dysregulated in the presence of high ironload (Song et al., 2018).

Animal models suggest that liver hepcidin has important consequences in brain pathologies, such as brain hemorrhage/ischemia. During brain hemorrhage/ischemia liver hepcidin is upregulated, which is accompanied with increased global brain iron content after peak levels of hepcidin (Xiong et al., 2016). Importantly, hepcidin knockout decreases brain iron content in models with brain hemorrhage, but not in physiological conditions (Tan et al., 2016; Xiong et al., 2016). Xiong et al. (2016) study found a significantly lower brain iron content in hepcidin knockout rodents compared to normal rodents during intracerebral hemorrhage (ICH). In addition, brain markers of oxidative stress and brain water content were lower in hepcidin knockout rodents compared to the brain of normal rodents during ICH. Finally, hepcidin knockout rodents performed better than their normal counterparts during cognitive tests. Together these data suggest that the presence of hepcidin impacts brain iron content and function during ICH. Data from human studies seem to complement findings from animal studies because they suggest that systemic hepcidin could contribute to increased brain damage during ICH. This observation is based on the existence of the correlation between serum levels of hepcidin and clinical scores that predict poor outcome in these patients (Xiong et al., 2015). This is the reason why Xiong et al. (2016) conclude that hepcidin affects brain iron efflux during ICH, but how could this action occur is unknown. It is interesting to notice that hepcidin has opposite effects on brain iron content during brain iron-overload that occurs without inflammation (Du et al., 2015; Gong et al., 2016). Du et al. (2015) study showed that the use ad-hepcidin after injection of holotransferrin into left carotid artery results in reduced brain iron content compared to rodents not treated with ad-hepcidin (Du et al., 2015). *In vitro* results from this study showed that ad-hepcidin and hepcidin peptide reduced brain iron uptake by decreasing TFR1, DMT1, and FPN expression in brain endothelial cells. So, how could hepcidin presence during ICH cause opposite results? During ICH brain iron is increased even many weeks after the insult (Dietrich and Bradley, 1988; Wu et al., 2003; Hua et al., 2006; Liu et al., 2016). The increase in brain iron content in this setting might occur because during BBB disruption iron leakage to brain parenchyma cannot be accompanied with reactive iron efflux since hepcidin blocks excess iron from getting out of brain cells. In this respect, hepcidin further enhances the effect of inflammatory signals on cellular iron accumulation (Sansing et al., 2011; Urrutia et al., 2013; Zhou et al., 2014; Xiong et al., 2016; Goldstein et al., 2017). This occurs because during brain inflammation, cytokines, and hepcidin have agonistic effects in suppressing cellular iron efflux (Urrutia et al., 2013; Xiong et al., 2016; Zhao Y. et al., 2018). In addition, inflammatory signaling overrides the blocking effect of hepcidin on cellular iron uptake which creates an ideal environment that promotes brain iron overload by both, inflammatory signals and hepcidin (Urrutia et al., 2013; Du et al., 2015; Gong et al., 2016; Xiong et al., 2016; Zhou et al., 2017; Zhao Y. et al., 2018).

## Brain Hepcidin Expression and Mechanisms of Regulation

Local hepcidin expression in the brain has been an object of investigation of different studies. In physiological conditions hepcidin expression in brain structures and cells (neurons, glial cells, endothelial cells, epithelial cells of choroid plexus) has been consistently observed by *in vivo* studies in humans and rodents, albeit in low levels (Krause et al., 2000; Pigeon et al., 2001; Zechel et al., 2006; Wang et al., 2008, 2010; Hänninen et al., 2009; Raha et al., 2013; Wei et al., 2014; Farajdokht et al., 2015; Raha-Chowdhury et al., 2015; Graf et al., 2016; Li Y. et al., 2016; Pan et al., 2016; Tan et al., 2016; Lu et al., 2017; You et al., 2017; Zhang et al., 2017). Data from human and animal studies suggest that local hepcidin is more robustly expressed in pathophysiological states (Sun et al., 2012; Urrutia et al., 2013; Tan et al., 2016; Xiong et al., 2016; You et al., 2017; Zhang et al., 2017). Similar to other cells, hepcidin main target protein in brain cells is FPN, but also iron import proteins (Sun et al., 2012; Urrutia et al., 2013; Tan et al., 2016; Xiong et al., 2016; You et al., 2017; Zhang et al., 2017).

Hepcidin upregulation can be elicited by acute iron load in astrocytes and microglia, while in neurons this response seems to become more evident with higher doses of iron supplementation (Sun et al., 2012; Urrutia et al., 2013; Simpson et al., 2015). On the other hand, according to Burkhart et al. (2016) hepcidin expression in the brain stem is scarce and probably has no effect during physiological conditions. It has to be mentioned that Burkhart et al. (2016) model of study has many differences compared to other authors. Burkhart et al. (2016) study did not include a bichamber model of BBB as compared to others, which imitates the cellular environment of brain endothelial cells and adjacent astrocytes (McCarthy and Kosman, 2014; Simpson et al., 2015). Still, even authors of this study acknowledge that hepcidin has an important role in brain iron homeostasis during iron-overload and conditions associated with BBB disruption (Burkhart et al., 2016). Unfortunately, compared to liver hepcidin, we still do not know the detailed mechanistic aspects of iron-induced regulation of hepcidin expression in brain cells. It is interesting to notice that results from studies with BMP6 pretreatment experiments are similar to those that have used pretreatment with hepcidin in terms of protection of brain cells from oxidative stress. But, whether BMP6 can control hepcidin expression in response to ironload in brain cells is still not known (Wang et al., 2001; Urrutia et al., 2017).

**FIGURE 1:**
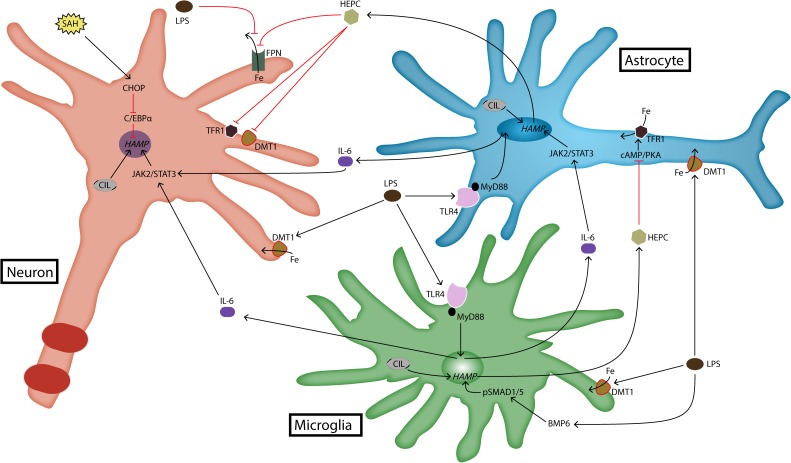
Expression and regulation of hepcidin in brain cells. Brain hepcidin expression is upregulated during inflammation. LPS is a product of inflammation that activates TLR4 receptor. Activated TLR4 induces the production of cytokines such as IL-6, which then goes on to upregulate hepcidin expression via JAK2/STAT3 pathway. Recently, it has been shown that LPS can induce hepcidin expression in microglia through BMP/SMAD pathway as well. LPS is a strong stimulus for iron import, because it induces upregulation of DMT1. Another pathway that can induce hepcidin expression in neurons is realized through CHOP and C/EBPα molecules. This occurs during SAH. In any case, most of hepcidin is produced by glial cells during inflammatory conditions. It acts on target cells by degrading FPN, which reduces iron export out of brain cells. Hepcidin also downregulates iron import proteins, DMT1 and TFR1. In astrocytes this action is realized through cAMP/PKA pathway. BMP6, bone morphogenetic protein 6; cAMP, cyclic adenosine monophosphate; C/EBPα, CCAAT/enhancer-binding protein α; CHOP, C/EBP homologous protein; CIL, cellular iron load; DMT1, divalent metal transporter 1; Fe, iron; FPN, ferroportin; *HAMP*, hepcidin antimicrobial peptide gene; HEPC, hepcidin; IL-6, interleukin-6; JAK2, janus kinase 2; LPS, lipopolysaccharide; MyD88, myeloid differentiation primary response 88; PKA, protein kinase A; SAH, subarachnoid hemorrhage; SMAD1/5, s-mothers against decapentaplegic 1/5; STAT3, signal transducer and activator of transcription 3; TFR1, transferrin receptor 1; TLR4, toll-like receptor 4.

On the other hand, there is quite robust data concerning hepcidin regulation in the brain via an inflammatory cascade which involves lipopolysaccharide (LPS), toll-like receptor 4 (TLR4), IL-6, STAT3 molecules (**Figure 1**). The magnitude of cell-specific response to LPS is highest in glial cells and lowest in neurons (Urrutia et al., 2013; You et al., 2017). Furthermore, TLR4 as the main membrane receptor for LPS is more consistently present in microglia (Trotta et al., 2014). In addition, studies suggest that IL-6 (which is a downstream molecule induced by LPS activity) is the mediator of hepcidin upregulation in the brain during inflammation (Zhang et al., 2017). Knockdown of IL-6 during brain inflammation reduces significantly the extent of STAT3 phosphorylation and resultant hepcidin upregulation and FPN downregulation (Zhang et al., 2017). Furthermore, IL-6 activity is associated with overexpression of DMT1, though this effect is not as robust as the consequences in reduction of iron export through FPN (Zhang et al., 2017). Urrutia et al. (2013) and Zhang et al. (2017) have revealed that although the effect of IL-6 in brain hepcidin expression is robust, it is not the only cytokine through which LPS regulates hepcidin. Intriguingly, FPN downregulation during inflammation is not related only to STAT3 or hepcidin levels, but due to an unknown IL-6 dependent pathway (Zhang et al., 2017). This finding is in-line with the suggestion that inflammatory signaling independent of hepcidin expression is the main culprit behind downregulation of FPN in brain cells. Cellular analysis shows that in an inflammatory setting the most evident changes in cellular iron load are observed in neurons and microglia, but less in astrocytes (Urrutia et al., 2013). This is probably due to the ability of astrocytes to facilitate iron transport in and out of their cellular environment (Xu H. et al., 2017). This would make sense if we take into account the general role of astrocytes as supporting cells that mediate transport and metabolism of different molecules.

Recently, two other pathways have been observed as inducers of brain hepcidin expression. This includes the canonical BMP/SMAD pathway, which is the classical pathway of hepcidin regulation in liver, and CCAAT/enhancer-binding protein (C/EBP) homologous protein (CHOP), which has also been involved in regulating liver hepcidin expression during endoplasmic reticulum (ER) stress (Oliveira et al., 2009). BMP/SMAD pathway has been shown to be involved in hepcidin expression in activated microglia in response to LPS, albeit in a reduced manner compared to IL-6/STAT3 pathway (Qian et al., 2014; Shin et al., 2018). On the other hand, CHOP pathway is involved in hepcidin expression in neurons, especially during brain hemorrhage. Similarly to liver, data suggest that CHOP regulates hepcidin expression in neurons via C/EBPα (Zhao J. et al., 2018). In this way, the picture of hepcidin regulation in the brain is unfolding, which will help in choosing the appropriate therapeutic strategies in different brain pathologies. Indeed, experiments with brain hepcidin knockout have resulted in decreased brain iron content in models with brain hemorrhage, while BMP/SMAD pathway suppression has recently been used in animal models to treat iron overload caused by hepcidin upregulation during brain ischemia (Ding et al., 2011; Tan et al., 2016; Shin et al., 2018). Similarly, CHOP silencing with siRNA reduces brain edema and improves neuronal function (Zhao J. et al., 2018). Also, TLR4 knockdown prevents brain iron accumulation during inflammation (Xiong et al., 2016).

## Dual Role of Hepcidin in Brain Pathologies; Implications for its Therapeutic Potential

It is evident that local and/or systemic hepcidin is induced during brain inflammation and iron load (Urrutia et al., 2013; Xiong et al., 2016). *In vivo* experiments during brain inflammation show that the use of iron chelation protects from brain iron overload, reduces microglial activation and improves cognitive functions in rodents by reducing levels of hepcidin and increasing levels of FPN in hippocampus (Li Y. et al., 2016; Pan et al., 2016). In addition, maternal diet and calorie restriction have been proposed as factors that offer neuroprotection by reducing brain iron load through suppression of brain hepcidin (Wei et al., 2014; Graf et al., 2016). But, it is interesting to notice that accumulating data suggest that hepcidin role in neuronal iron load and inflammation is ambiguous; hepcidin can protect from iron load, but also can cause iron load during inflammation. The reason for this duality seems to occur due to the timing of hepcidin action; studies show that pretreatment with hepcidin protects brain cells from iron load, while hepcidin induction during inflammation aggravates iron load (Xiong et al., 2016; Urrutia et al., 2017; Zhao Y. et al., 2018). Inflammation in the brain is a strong stimulus for the increase in iron import, which increases iron load. But, inflammation has a double effect on iron export; it blocks iron export via FPN inhibition through hepcidin-independent and hepcidin-dependent mechanisms in neurons and glial cells (Urrutia et al., 2013; Zhang et al., 2017). The double effect of inflammation in blocking iron export enhances the cellular iron load in the brain even further. Previous studies have indicated that LPS can indeed affect FPN expression independently of cytokines, though cellular specific differences do exist (Liu et al., 2005; Deschemin and Vaulont, 2013). Cellular specific reactions to inflammatory stimuli have revealed that astrocytes and microglia (compared to neurons) react more strongly to LPS by increasing their hepcidin expression and decreasing FPN expression (Urrutia et al., 2013; Ma et al., 2018). Isolated neurons do not show signs of robust increase in hepcidin levels and FPN downregulation in reaction to inflammatory stimuli (Ma et al., 2018; Puy et al., 2018). But, when neurons are stimulated with high dose of inflammatory cytokines their hepcidin expression is increased significantly (Urrutia et al., 2013). This environment is created when neurons are co-cultured with microglia and astrocytes (You et al., 2017). In this scenario, prime responders to LPS are microglia, which through IL-6 act on astrocytes to increase hepcidin expression. Then, hepcidin acts on neurons in a paracrine manner to reduce FPN expression (You et al., 2017). It has to be mentioned that microglia can also affect neuronal hepcidin expression via LPS (Qian et al., 2014; Li Y. et al., 2016). So, the present picture suggests that during inflammation astrocytes are responsible for directing iron influx from plasma into the brain, where this iron is deposited in neurons via microglial and astrocyte activity, which can increase neuronal oxidative damage and even cause neurodegeneration.

**FIGURE 2:**
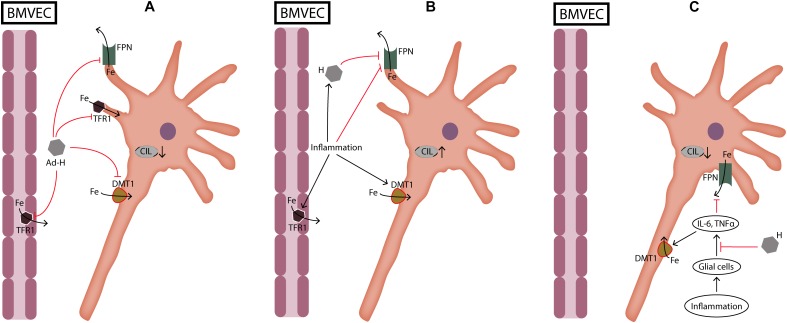
Dual role of hepcidin during neuronal cellular iron load and inflammation. **(A)** During cellular iron load not caused by inflammation hepcidin protects neurons by reducing iron transport through BMVEC and by reducing iron import into neurons. **(B)** During inflammation hepcidin production induced by cytokines is deleterious for neurons because it increases iron load by inhibiting iron export through FPN. On the other hand, inflammation increases iron import into cells which further aggravates cellular iron load. **(C)** When glial cells are pretreated with hepcidin, cellular iron load is decreased because hepcidin suppresses the release of cytokines from glial cells. Ad-H, ad-hepcidin; BMVEC, brain microvascular endothelial cells; CIL, cellular iron load; DMT1, divalent metal transporter 1; Fe, iron; FPN, ferroportin; IL-6, interleukin-6; TFR1, transferrin receptor 1; TNFα, tumor necrosis factor α.

On the other hand, hepcidin pretreatment has the potential to relieve the damage caused by inflammatory signaling. This happens because hepcidin pretreatment downregulates IL-6 and tumor necrosis α (TNFα) expression in astrocytes and microglia (Urrutia et al., 2017) (**Figure 2**). Treatment with hepcidin in these conditions protects neurons from oxidative stress (Urrutia et al., 2017). In addition, treatment with ad-hepcidin in non-inflammatory conditions protects neurons from iron load (Du et al., 2015; Gong et al., 2016; Zhou et al., 2017). The protective role of hepcidin occurs due to its effect in inhibiting iron transport through BMVEC and through its effects in reducing iron import in neuronal cells (Du et al., 2015; Gong et al., 2016; Zhou et al., 2017). These data show that timing of hepcidin treatment and presence of inflammation dramatically influences hepcidin actions in brain cells. It seems that hepcidin pretreatment primes the brain cellular milieu against the stimulative effects of inflammatory signals in increasing brain iron load (Urrutia et al., 2017). Previous studies with cultured macrophages have revealed that hepcidin-FPN interaction induces suppressor of cytokine signaling 3 (SOCS3) activity, which as its name suggest, is a suppressor of cytokine signaling during inflammation (De Domenico et al., 2010). Also, human studies have shown that levels of hepcidin are related with the inflammatory response (Burté et al., 2013). It would be interesting for future studies to reveal if beneficial effects of pretreatment with hepcidin can be sustained with increasing levels of inflammation.

## Role of Inflammation and Iron Load in Neurodegenerative Diseases

Pathogenesis of neurodegenerative diseases is complex and still not entirely understood. But, in recent years accumulating evidence are linking neurodegenerative processes with iron metabolism and neuroinflammation. Iron dysmetabolism is an early feature of Alzheimer’s disease (AD) and in isolated nuclei in Parkinson’s disease (PD) (Nunomura et al., 2001; Smith et al., 2010; Guan et al., 2017). In AD, levels of ferritin in cerebrospinal fluid (CSF) are predictors of worsening cognitive performance and are also correlated with apolipoprotein E (ApoE) CSF levels and AD risk allele 𝜀4 (Ayton et al., 2015). Similarly, higher iron deposition in substantia nigra (SN) of AD patients makes them more prone to develop PD (Brar et al., 2009). Use of compounds with moderate iron-binding potency reduces levels of α-synclein and protects neurons from oxidative damage in PD (Finkelstein et al., 2017). This effect is realized by restoring the function of FPN as a cellular iron exporter (Finkelstein et al., 2017). Iron load as a risk factor in PD has been further confirmed in a meta-analysis, which has identified the presence of iron overload in SN of PD patients (Wang et al., 2016). It seems that in PD iron overload is generally more present in advanced stages of the disease compared to AD (Wang et al., 2016; Guan et al., 2017). In patients with mild and moderate PD there is also evidence of systemic perturbations of iron metabolism, with ferritin and malondialdehyde (MDA) serum levels (marker of oxidative damage) serving as significant biomarkers of PD (De Farias et al., 2017). On the other hand, it has to be mentioned that the most affected brain structure in PD, that is SN, is characterized with iron overload and significant damage in early stages of the disease (Ziegler et al., 2013; Guan et al., 2017). This would imply that iron dysmetabolism in SN in PD could occur due to a similar pathogenic mechanism observed in AD. It is interesting to notice that APP dysfunction (which is a hallmark of AD) induces dementia-like symptoms in PD (Lei et al., 2012). Importantly, APP dysfunction in AD results in its inability to stabilize the function of FPN as a cellular iron exporter, which would affect intracellular iron load (Duce et al., 2010).

Similar to iron load, inflammation is also being recognized as an important pathogenic marker of neurodegenerative diseases. It is observed early in AD and PD, often bearing evidence of peripheral (intestinal) aberrant response to different stimuli (Qin et al., 2016; Houser and Tansey, 2017; Raj et al., 2017; Gelders et al., 2018; Gurel et al., 2018; King et al., 2018; Steeland et al., 2018). This means that in AD and PD the pathogenic process is initiated and “fueled” by immunologic disturbances occurring in the gastrointestinal tract. In relation to this scenario are data coming from studies with the TNF receptor (TNFR), which have caught attention for their importance in neurodegenerative diseases. TNF and Aβ-induced inflammation is significantly reduced via TNFR1 loss (Steeland et al., 2018). It seems that TNF and Aβ oligomers act as agonists on TNFR1 to activate microglial cells. In addition, TNFR1 loss protects brain cells from propagation of peripheral inflammatory reaction by restoring blood-CSF barrier functionality (Steeland et al., 2018). In addition, direct evidence from human studies with inflammatory bowel disease (IBD) suggests that early TNF inhibition dramatically reduces the incidence of PD (Peter et al., 2018). TNF involvement in PD is further strengthened by the increased risk of early onset of PD in patients with higher expression of TNF (Lindenau et al., 2017). On the other hand, role of inflammation in AD and PD is characterized with subtle but important differences between these conditions; *in vivo* models with mice have shown that Aβ oligomer accumulation in AD is related to TLR4 activity, which is in contrast with α-synuclein accumulation in PD, where the role of TLR4 is low or inexistent (Noelker et al., 2013; Forloni et al., 2016; Calvo-Rodríguez et al., 2017). Also, microglial activation is somewhat more prominent with AD related oligomers than with PD related oligomers (Forloni et al., 2016). Furthermore, in PD, microglial activity is accompanied with increased number of lymphocytes in damaged brain nuclei (Wang et al., 2015). These data suggest distinct inflammatory routes of activation by pathogenic oligomers in AD and PD.

Alzheimer’s disease and PD are diseases that increase in prevalence with age. It is believed that the ability of microglia and astrocytes to secure the homeostatic equilibrium for neurons is reduced with aging. The homeostatic disequilibrium might be a result of the genetic factors that are related with increased activity of inflammatory signals, decreased ability of microglia to clear oligomer accumulation and other factors (Heppner et al., 2015; Lindenau et al., 2017; Hansen et al., 2018). “Pathogenic” microglia release cytokines which are responsible for neuronal damage, partly by inducing iron load. In experiments with cultured neurons, accumulation of pathological Aβ oligomers produces significant neurotoxic effects when neurons are co-cultured with glial cells (Urrutia et al., 2017). This shows that glial cell activity is an important factor in potentiating the toxic effect of Aβ oligomers. In order to protect themselves from cellular damage neurons increase ferritin depots, but the accumulating iron is very reactive and once it accumulates in sufficient amount it will cause oxidative damage. Iron load initiates a vicious cycle of neuronal damage, caused by the ability of excess iron to accelerate oligomer toxicity (Rottkamp et al., 2001; Wan et al., 2017).

Although evidence for the role of inflammation and resultant iron load in neurodegenerative diseases is accumulating, it is of therapeutic importance to explain how do these pathogenic factors change the dynamic of cellular biochemistry. Data from animal models show that aging creates a favorable milieu for neuronal damage via iron load. In rats, levels of DMT1 and hepcidin are increased, while levels of FPN do not change (Wang et al., 2010; Lu et al., 2017). This would indicate that cellular iron load with aging occurs due to increased iron import. Increased cellular iron stimulates hepcidin expression, which is not able to control FPN expression. It is interesting to notice that FPN is downregulated in aged rats with APP knockout (Belaidi et al., 2018). Furthermore, APP knockout increases the rate of brain iron accumulation that occurs with aging. This means that the preservation of cellular iron efflux is important for slowing age-related brain iron overload. In brains of AD patients and mice hepcidin expression is located in damaged neurons and blood vessels of amyloid plaques (Raha et al., 2013). On the other hand, similar to changes occurring with age, we observe increased DMT1 expression, but also co-localization of DMT1 with amyloid plaques in AD patients and in animal models with AD (Zheng et al., 2009). Animal models suggest that DMT1 expression is directly linked with the metabolism of pathogenic peptides that accumulate during AD (Zheng et al., 2009). Furthermore, DMT1 silencing increases cell viability in AD (Zheng et al., 2009). Similarly, in human patients, animal models and cell cultures with PD, DMT1 is also upregulated and related to oxidative stress (Salazar et al., 2008; Chen et al., 2015). Consequently, blocking DMT1 and hepcidin improves cell viability, while FPN upregulation rescues neuronal function in cultured cells and mice brain with PD (Chen et al., 2015; Xu et al., 2016; Xu Q. et al., 2017). So, at least in animal models with neurodegenerative disease, suppression of iron import and stimulation of iron export (via hepcidin manipulation) ameliorates neuronal degeneration.

In other diseases accompanied with neurodegeneration (Huntington disease, amyotrophic lateral sclerosis) iron deposition is also increased in different brain structures (Chen et al., 2013; Muller and Leavitt, 2014; Szeliga et al., 2016; Moreau et al., 2018). Changes in expression of iron protein carriers and the therapeutic potential of iron chelation evoke striking similarities with AD and PD (Salazar et al., 2008; Zheng et al., 2009; Chen et al., 2013; Belaidi and Bush, 2016; Szeliga et al., 2016; Moreau et al., 2018). It is speculative to suggest that the role of hepcidin in neurodegenerative diseases might be of primary importance, due to the dominating role of innate immune system in the pathophysiology of these diseases. Hepcidin has been evidenced quite robustly as part of our innate immune response, which is helpful in acute situations, because it controls iron availability to pathogens, but also it can regulate actions of inflammatory cytokines (De Domenico et al., 2010; Armitage et al., 2011; Burté et al., 2013). But, during chronic innate immune activity the negative feedback control between hepcidin and inflammation observed in acute situations turns into an agonistic relationship which will eventually deteriorate brain cell damage.

## The Rationale for Using Hepcidin Therapeutics in Neurodegenerative Diseases

Stimulation and inhibition of hepcidin has been used in cultured cells and animal models to ameliorate brain damage. The rationale is evident; hepcidin affects FPN and iron import proteins, which means its manipulation can control cellular iron load. Furthermore, it has been shown that decreased activity or expression of FPN promotes neuronal damage (Song et al., 2010; Yang et al., 2011; Crespo et al., 2014). In animal models with PD neurodegeneration is associated with increased microglial activity and FPN downregulation (Zhang et al., 2014). In addition, human trials with iron chelators have shown promise in retarding the progress of neuronal damage (Crapper McLachlan et al., 1991; Martin-Bastida et al., 2017; Moreau et al., 2018). But, long-term results of this therapy are still unknown and pending future trials (Adlard and Bush, 2018).

In animal models with brain inflammation and increased oxidative stress, direct and indirect suppression of local and systemic hepcidin offers neuroprotection (Chen et al., 2015; Li W.-Y. et al., 2016; Pan et al., 2016; Xiong et al., 2016). But, in conditions with lack of inflammation, the use of ad-hepcidin has also beneficial effects in neuronal function, probably due to hepcidin ability to protect neurons during iron overload via suppression of cellular iron import proteins. Still, there exist practical issues concerning the use of delivery methods for hepcidin in these scenarios. In animal models this has been done via intracerebroventricular injections, but the feasibility of this method in humans is unknown (Gong et al., 2016; Urrutia et al., 2017). Recent advances suggest that nanotechnology could make an important breakthrough in treating brain diseases with targeted delivery of peptides (Goldsmith et al., 2014; Silva Adaya et al., 2017).

Based on the accumulating evidence about the role of inflammation on cellular iron content, it is reasonable to assume that neuroprotection can be achieved by blocking the inflammatory pathways through already established drugs. One such drug is acetylsalicylic acid, which has already been used in cultured cells to protect neuronal cells and microglia from inflammation-induced damage (Li W.-Y. et al., 2016; Huang et al., 2018) (**Table 1**). The protective effect of acetylsalicylic acid is realized through increase in iron export in neuronal cells (Huang et al., 2018), while in microglia it reduces iron import and increases iron export (Xu et al., 2015).

**Table 1:** Therapeutic potential of the manipulation of hepcidin and its target proteins in neurodegeneration.

Condition	Method of study	Main results	Reference
Brain inflammation	Effects of hepcidin suppression in brains of mice with intracerebral hemorrhage and in cultured cells	Liver hepcidin knockout reduces brain damage Treatment with hepcidin aggravates brain damage Suppression of brain hepcidin by blocking inflammatory stimuli reduces brain damage	Xiong et al., 2016
Brain inflammation	Effects of hepcidin suppression in rat brains with brain ischemia	Knockdown of brain hepcidin reduces iron load by increasing FPN expression	Ding et al., 2011
Brain inflammation	Effects of hepcidin suppression in rat brains with subarachnoid hemorrhage	Hepcidin injections increase cellular apoptosis Knockdown of brain hepcidin ameliorates brain damage	Tan et al., 2016
Brain inflammation	Effects of aspirin on cytokine actions in cultured microglial cells	Aspirin protects cultured microglial cells from LPS-induced damage Aspirin induces FPN upregulation and reduces ferritin levels in microglial cells treated with LPS Aspirin suppresses IL-6, TNFα and hepcidin expression in microglial cells treated with LPS	Xu et al., 2015
Brain inflammation	Effects of aspirin on cytokine actions in cultured microglial cells	Aspirin protects cultured microglial cells from LPS-induced damage Aspirin suppresses hepcidin expression in microglial cells treated with LPS Aspirin suppresses IL-6 expression in microglial cells treated with or without LPS	Li W.-Y. et al., 2016
Brain inflammation	Effects of aspirin on cytokine actions in cultured neuronal cells	Aspirin protects neuronal cells from inflammation-induced signaling Aspirin increases FPN expression and decreases hepcidin and ferritin levels in neuronal cells treated with IL-6	Huang et al., 2018
Brain ischemia/inflammation	Effects of L-LYC on cytokine actions in rat brains with ischemia/inflammation	L-LYC protects brain cells during brain ischemia/inflammation L-LYC reduces hepcidin expression and increases FPN expression in neuronal cells L-LYC reduces IL-6 expression and hepcidin expression in rat brains	Zhao Y. et al., 2018
Brain inflammation	Effects of dalteparin on IL-6 and hepcidin in mouse model with chronic mild stress	Dalteparin protects from brain iron load Dalteparin reduces brain hepcidin expression Dalteparin reduces serum IL-6	Farajdokht et al., 2015
Brain inflammation/ischemia	Effects of tanshinone IIA on iron-related proteins in rat brains and cultured neurons	Tanshinone IIA has neuroprotective properties during brain ischemia Tanshinone IIA downregulates TFR1, DMT1, while it upregulates FPN expression in rat brains Tanshinone IIA downregulates TFR1, DMT1, while it upregulates FPN expression in cultured neurons	Yang et al., 2011
Brain amyloid-β toxicity	Effects of hepcidin pretreatment in mice brain and cultured mice brain cells	Hepcidin pretreatment reduces IL-6 and TNFα expression in astrocytes and microglia treated with amyloid-β aggregates Hepcidin pretreatment reduces astrocyte and microglia activation in mice brain Hepcidin pretreatment protects from amyloid-β induced oxidative damage in mice brain Hepcidin pretreatment suppresses the ability of microglia and astrocytes treated with amyloid-β to induce oxidative damage in neurons	Urrutia et al., 2017
Alzheimer’s disease	Effects of DMT1 silencing in neuronal cell cultures	DMT1 silencing reduces cellular iron load DMT1 silencing reduces the expression of APP and Aβ peptide DMT1 silencing increases cell viability	Zheng et al., 2009
Parkinson’s disease	Effects of EGCG on neuronal cultured cells with Parkinson’s disease model induced by 6-OHDA	EGCG protects neurons from cellular death and increases cell viability EGCG decreases DMT1, hepcidin, ferritin expression, while it increases FPN expression	Chen et al., 2015
Parkinson’s disease	Effect of hepcidin on neuronal cultured cells with Parkinson’s disease model induced by 6-OHDA	Hepcidin knockdown with siRNA protects neurons from cytotoxic effects of 6-OHDA	Xu et al., 2016
Brain iron-overload without inflammation	Effects of ad-hepcidin in rat brains and cultured cells with iron overload	Ad-hepcidin reduces brain iron overload Ad-hepcidin reduces iron uptake proteins (DMT1, TFR1) and iron release protein (FPN) in cultured BMVEC Ad-hepcidin reduces iron uptake proteins (DMT1, TFR1) and iron release protein (FPN) in cultured neurons	Du et al., 2015
Brain iron-overload without inflammation	Effects of ad-hepcidin in rat brains with iron overload	Ad-hepcidin reduces iron load in iron overload brains	Gong et al., 2016
Brain iron-overload without inflammation	Effects of ad-hepcidin in cultured neurons during hemin-induced injury	Ad-hepcidin reduces cellular iron content in cultured neurons treated with hemin Hepcidin downregulates the levels of DMT1, TFR1, and FPN in cultured neurons treated with hemin	Zhou et al., 2017

*6-OHDA, 6-hydroxydopamine; APP, amyloid precursor protein; BMVEC, brain microvascular endothelial cells; DMT1, divalent metal transporter 1; EGCG, epigallocatechin-3-gallate; FPN, ferroportin; IL-6, interleukin-6; L-LYC, liposomal lycopene; LPS, lipopolysaccharide; siRNA, small interfering RNA; TFR1, transferrin receptor 1; TNFα, tumor necrosis factor α.*

As it was previously mentioned heparins can affect liver hepcidin expression. One such heparin, called dalteparin, has been administered via intraperitoneal injections in mice to suppress brain hepcidin expression during inflammatory conditions (Farajdokht et al., 2015). Dalteparin can also suppress serum IL-6 levels in these conditions, which means that its beneficial properties in reducing increased brain iron during inflammation could be due to suppression if systemic inflammation and brain hepcidin. Still the cause/effect relationship of dalteparin in the brain has not been studied thoroughly in terms of its impact on brain iron metabolism via hepcidin regulation. Future studies should also explain whether dalteparin-induced brain hepcidin suppression is dose-dependent, because of the side effects of this drug on brain homeostasis (Perry et al., 2010).

Another therapeutic option that has been used is based on methods of delivery that increase the efficiency of neuroprotective substances. One example is the use of liposomes, which serve as particles that deliver drugs to target cells, whilst keeping intact the stability of the substance that is being used for therapeutic purposes. Liposomal lycopene has been delivered in rat brains to reduce oxidative stress and ameliorate apoptosis caused by ischemia. This effect is at least partly attributed to the ability of lycopene to reduce hepcidin expression in astrocytes via its suppressive effect on IL-6 signaling (Zhao Y. et al., 2018).

## Conclusion

Hepcidin is emerging as an important peptide for brain (patho)physiology. The source of hepcidin in the brain is local and probably systemic. It is believed that systemic hepcidin can pass through BBB, especially during BBB leakage (for example during inflammation). Neurons, glial cells, endothelial cells, and other brain cells may express hepcidin, although basal levels of brain hepcidin are low. They increase significantly during inflammation and most likely during brain iron load. The local molecular regulation of brain hepcidin expression is unfolding, while studies suggest that it bears resemblance with liver hepcidin regulation. Still, the details of this regulation await further clarification from future studies. Hepcidin produced by the brain cells in pathological conditions may limit iron transport through BMVEC, and thereby also limit neuronal iron load. High iron load is detrimental for neuronal function. In addition, high iron load can also affect glial cell activity by turning them into cells that promote neuronal damage. The observation of robust production of hepcidin by glial cells compared to neurons during changes in brain homeostasis is in-line with the already established role of glia as supportive, regulatory and protective cells in the brain milieu. During inflammatory conditions hepcidin promotes neuronal iron load, while the opposite occurs in non-inflammatory conditions. This observation is based on studies that have revealed the neuroprotective nature of hepcidin suppression during inflammation. This occurs due to the increase in iron import caused by inflammation, which is further exacerbated by FPN downregulation caused by hepcidin-dependent and hepcidin-independent mechanisms. On the other hand, use of ad-hepcidin during neuronal iron load (not caused by inflammation) protects these cells from oxidative stress. Intriguingly, hepcidin pretreatment also protects neurons from the deleterious effects of inflammation. It seems that pretreatment with hepcidin blocks the initiation of inflammation-induced biochemical cascade inside brain cells. This effect of hepcidin is not without precedent, because it has been observed in mice and human studies. Unfortunately, therapeutic implications of this physiological effect of hepcidin have not been studied in details, despite its potential crucial importance in brain and other pathologies. Still, pharmacologic manipulation of hepcidin is emerging as e new therapeutic territory in neurodegenerative diseases. Animal models have shown that the use of ad-hepcidin and suppression of hepcidin protects brain cells in models of neurodegenerative diseases. This has been further confirmed with the use of standard anti-inflammatory drugs such as acetylsalicylic acid or lycopene.

Finally, considering the ever increasing importance of hepcidin for brain physiology, it is paramount for future studies to examine models with different cell specific hepcidin knockout in AD and PD that will reveal the temporal changes of hepcidin, but that will also define whether hepcidin is of primary importance for the pathophysiology of neurodegenerative diseases.

## Author Contributions

The author confirms being the sole contributor of this work and has approved it for publication.

## Conflict of Interest Statement

The author declares that the research was conducted in the absence of any commercial or financial relationships that could be construed as a potential conflict of interest.
